# Are orthobiologics a plausible therapeutic option for patello-femoral pathology? A systematic review of clinical and radiological outcomes

**DOI:** 10.3389/fbioe.2026.1842090

**Published:** 2026-06-12

**Authors:** Carlotta Franceschi, Yazan Abu Salem, Pietro Conte, Giuseppe Anzillotti, Berardo Di Matteo, Marina Lipina, Alexey Lychagin, Eugene Kalinsky, Elizaveta Kon

**Affiliations:** 1 IRCCS Humanitas Research Hospital, Rozzano, Milan, Italy; 2 Department of Biomedical Sciences, Humanitas University, Milan, Italy; 3 Joint Stock Company, Medsi Group, Moscow, Russia; 4 Department of Traumatology, Orthopaedics, Sechenov First Moscow State Medical University, Moscow, Russia

**Keywords:** knee, mesenchymal stromal cell, orthobiologic, patella, patello-femoral, platelet-rich plasma, regenerative medicine

## Abstract

**Introduction:**

Even though orthobiologics have been extensively studied in the treatment of tricompartmental knee osteoarthritis (OA), evidence specifically addressing their role in the management of patello-femoral pathology remains limited. Thus, the aim of the present systematic review is to critically analyze and synthesize the available clinical and, where reported, radiological evidence on the safety and the therapeutic efficacy of platelet-rich plasma (PRP)- and mesenchymal stromal cell (MSC)-based products in the treatment of patello-femoral chondropathy (PFC) and patello-femoral osteoarthritis (PFOA).

**Materials and methods:**

A comprehensive search of four medical electronic databases (PubMed, Embase, Google Scholar, and Cochrane) was conducted to identify randomized controlled trials, prospective or retrospective studies on humans evaluating the use of PRP- or MSC-based products in the treatment of PFC and PFOA.

**Results:**

A total of nine eligible studies were included; of them, four investigated PRP-based therapies and five evaluated MSC-based products. Despite the generally poor methodological quality of the existing body of evidence, a consistent trend towards a clinical and, where reported, radiological improvement was observed across the included studies, with between-group comparisons confirming the clinical superiority of both PRP- and MSC-based approaches compared to the controls.

**Conclusion:**

Even though the available clinical and, where reported, radiological evidence remains limited and methodologically heterogeneous, PRP- and MSC-based products may hold a therapeutic potential for PFC and PFOA, extending beyond their established application for tricompartmental knee OA.

**Systematic Review Registration:**

identifier CRD420261343230.

## 1 Introduction

Patello-femoral chondropathy (PFC) and patello-femoral osteoarthritis (PFOA) are a common source of persistent anterior knee pain and severe functional limitations in activities of daily living and sports (Gaitonde et al., 2019), affecting young, high-demand individuals as well as elderly patients with tricompartmental knee osteoarthritis (OA) (Smith et al., 2018a; Hinman and Crossley, 2007; Garza-et al., 2024). This is most likely attributable to the distinctive biomechanical properties of the patello-femoral joint (Sherman et al., 2014; Mills and Hunter, 2014; Yu et al., 2019), which, indeed, exhibits a complex, multi-planar kinematics, high contact forces, and substantial shear stresses that, overall, contribute to make the articular cartilage of the patella and the femoral trochlea particularly susceptible to both traumatic and degenerative damage (Loudon, 2016; Wheatley et al., 2020). It is for this reason that patello-femoral pathology continues to represent a complex and demanding clinical entity, frequently associated with suboptimal long-term outcomes and, most importantly, a sustained impairment in patients’ quality of life (Smith et al., 2018b).

As for tricompartmental knee OA, conservative management – including patient education, activity modification, physiotherapy (Heintjes et al., 2015; Harvie et al., 2011; Crossley et al., 2002), patellar bracing (D’hondt et al., 2009), taping (Warden et al., 2008), local application of ice or heat, analgesics, e.g., oral non-steroidal anti-inflammatory drugs (NSAIDs) (Heintjes et al., 2004), and intra-articular injections of hyaluronic acid (HA) (Clarke et al., 2005) or corticosteroids (Heintjes et al., 2004) – should be the recommended initial approach (Saltychev et al., 2018; Petersen et al., 2017; Fulkerson, 2002; Kettunen et al., 2007; Collins et al., 2018). While these non-surgical strategies may provide a short-term symptomatic relief, their ability to target the underlying disease process remains limited. Surgery, ranging from arthroscopy, e.g., arthroscopic lavage and debridement, tibial tubercle osteotomy, and, eventually, patello-femoral arthroplasty, is usually reserved for refractory cases (McCarthy and Strickland, 2013; Kuwabara et al., 2022), yet outcomes remain variable, especially in young, high-demand patients (Sarda et al., 2011). For this reason, orthobiologics – that is, biological products derived from substances that are naturally present in the human body (Costa et al., 2025) – have been recently explored as an emerging regenerative medicine technology for the treatment of patello-femoral joint disorders, extending beyond their established application for tricompartmental knee OA in light of their well-documented immunomodulatory and anti-inflammatory properties (Perucca et al., 2023; Boffa et al., 2023; Boffa et al., 2021; Im, 2021). To date, the most promising alternatives in terms of pain alleviation, restoration of functional capacity, and potential tissue repair are platelet-rich plasma (PRP) and mesenchymal stromal cells (MSCs). PRP is an autologous platelet-rich blood derivative providing a concentrated source of endogenous platelet-derived growth factors that have been demonstrated to influence chondrocyte metabolism, modulate local inflammatory signalling pathways, and, ultimately, contribute to the maintenance of joint homeostasis (Marx, 2001). Similarly, MSCs are a heterogeneous population of multipotent stromal cells extracted from various tissue sources – usually bone marrow or adipose tissue – that have the potential to undergo chondrogenic differentiation *in vitro* and *in vivo* and, therefore, support the self-healing of the articular cartilage (Afanasyev et al., 2009).

Even though orthobiologics have been extensively studied in the treatment of tricompartmental knee OA (Laver et al., 2024; De et al., 2025; Kon et al., 2024), evidence specifically addressing their role in the management of patello-femoral pathology remains limited (Katz et al., 2024). Indeed, as discussed earlier, the patello-femoral compartment differs substantially from the tibio-femoral joint in terms of biomechanics and, consequently, disease behaviour, thus precluding the direct generalization of treatment outcomes. Moreover, the majority of studies on the topic that have been published in the literature so far exhibit substantial heterogeneity in terms of patient selection, types of products employed, injection protocols, outcome measures, and follow-up duration, resulting in the lack of specific indications for their application in the treatment of patello-femoral joint disorders (Laver et al., 2024; De et al., 2025; Kon et al., 2024). Thus, the aim of the present systematic review is to critically analyze and synthesize the available clinical and, where reported, radiological evidence on the safety and the therapeutic efficacy of PRP- and MSC-based products in the treatment of PFC and PFOA, clarifying their current clinical utility in the management of these increasingly prevalent and challenging conditions.

## 2 Materials and methods

The present systematic review was registered with the PROSPERO International Prospective Register of Systematic Reviews (registration number: CRD420261343230). A comprehensive search of four medical electronic databases (PubMed, Embase, Google Scholar, and Cochrane) was conducted on 17 December 2025 following the Preferred Reporting Items for Systematic Reviews and Meta-Analyses (PRISMA) guidelines. Boolean operators “AND” and “OR” were employed to systematically connect the selected search terms, which included “patello-femoral,” “patella,” “trochlea,” “orthobiologic,” “knee injection,” “platelet-rich plasma,” “PRP,” “mesenchymal stromal cell,” “MSC,” “bone marrow,” and “adipose tissue.” The following inclusion criteria were adopted to assess eligibility (Gaitonde et al., 2019): randomized controlled trials (RCTs), prospective or retrospective studies on humans (Smith et al., 2018a); written in English language (Hinman and Crossley, 2007); minimum five patients enrolled (Garza-et al., 2024); minimum 6 months of follow-up; and (Sherman et al., 2014) analyzing the available clinical and, where reported, radiological evidence on the safety and the therapeutic efficacy of PRP- or MSC-based products in the treatment of PFC and PFOA. We excluded papers written in languages other than English, *in vitro* studies, animal studies, narrative or systematic reviews and metanalyses, case reports or mini case-series (with fewer than five patients enrolled), and articles for which access to the full text was restricted.

**FIGURE 1:**
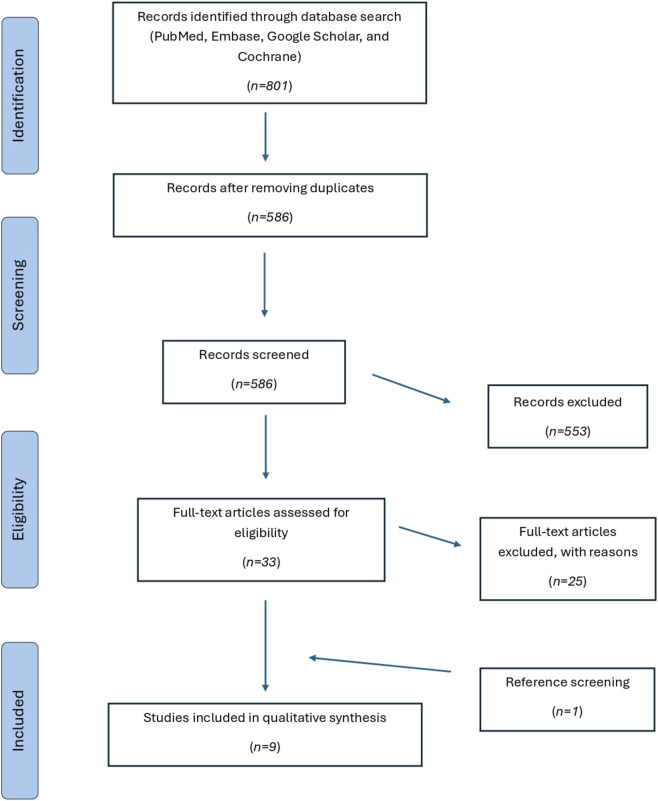
PRISMA flowchart illustrating the screening and selection process.

A total of 801 potentially eligible studies were identified through our database search. Following an initial title and abstract screening, the full texts of 33 articles were carefully analyzed and, after an in-depth evaluation, 25 were excluded because they were not related to the knee joint, did not specifically focus on patello-femoral joint disorders, included fewer than five cases, or reported an insufficient follow-up duration. Lastly, the reference list of the retrieved articles was further screened using the same inclusion and exclusion criteria previously outlined to identify other potentially relevant studies, resulting in the inclusion of one additional paper. A PRISMA flowchart of the screening and selection process is illustrated in Figure 1.

The screening process was performed independently by two investigators (CF and YAS), while data verification and analysis were supervised by two additional reviewers (GA and PC). Two senior investigators (BDM and EK) were responsible for resolving any discrepancies between the two afore-mentioned reviewers by reaching a consensus. Risk of bias and quality assessment were independently conducted by two authors (CF and YAS) following the Cochrane Risk of Bias 2.0 (RoB-2) Tool (Sterne et al., 2019) and the modified Coleman Methodology Score (CMS) introduced by Kon et al. (2009) for RCTs and prospective and retrospective studies, respectively.

## 3 Results

According to the above-mentioned inclusion and exclusion criteria, we identified a total of nine eligible studies. Four studies (Örsçelik et al., 2020; Cobianchi et al., 2021; Van et al., 2021; Ostojic et al., 2024) investigated the use of PRP-based therapies in the treatment of PFC and PFOA and five studies (Pintat et al., 2017; Guimarães et al., 2018; Vasso et al., 2022; Silvestre et al., 2023; Zhang et al., 2024) evaluated the use of MSC-based products in the treatment of PFOA (but Zhang et al. (2024), who included patients affected by multi-compartmental knee OA). PRP-based interventions included PRP and autologous protein solution (APS), whereas MSC-based products included stromal vascular fraction (SVF), bone marrow-derived mononuclear cells (BMMCs), micro-fragmented adipose tissue (MFAT), and bone marrow concentrate (BMC). Study designs were as follows: two RCTs (Örsçelik et al., 2020; Zhang et al., 2024), two retrospective case-control studies (Cobianchi et al., 2021; Silvestre et al., 2023), one prospective case series (Van et al., 2021), two prospective cohort studies (Ostojic et al., 2024; Pintat et al., 2017), one prospective pilot study (Guimarães et al., 2018), and one retrospective case series (Vasso et al., 2022). Sample sizes ranged from 8 to 96 participants.

### 3.1 Risk of bias assessment

**FIGURE 2:**
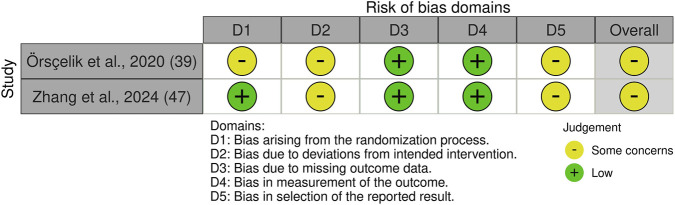
Risk of bias assessment of randomized controlled trials performed with the Cochrane Risk of Bias 2.0 Tool.

For the two RCTs included in the present systematic review, risk of bias assessment was conducted using the Cochrane RoB-2 Tool (Sterne et al., 2019). The RCT by Örsçelik et al. (2020) was rated as having some concerns overall due to issues arising from the randomization process and deviations from the intended intervention and in the selection of the reported results. Similarly, the RCT by Zhang et al. (2024) was judged to have some concerns overall due to uncertainties arising from deviations from the intended intervention and in the selection of the reported results. The results of the quality assessment are summarized in Figure 2 for each study.

For the remaining seven non-RCTs (Cobianchi et al., 2021; Van et al., 2021; Ostojic et al., 2024; Pintat et al., 2017; Guimarães et al., 2018; Vasso et al., 2022; Silvestre et al., 2023), risk of bias assessment was performed with the modified CMS introduced by Kon et al. (2009). The mean total score was 46/100, suggesting a generalized poor methodological quality for the included studies. A detailed summary of the quality assessment is illustrated in Table 1 for each study.

**TABLE 1:** Risk of bias assessment of non-randomized controlled trials performed with the modified Coleman Methodology Score introduced by Kon et al.

Publication	Study size (0–10)	Mean follow-up (months) (0–10)	Number of procedures (0–10)	Study design (0–15)	Description of the primary procedure (0–5)	Description of the post-operative rehabilitation (0–5)	MRI outcomes (0–10)	Histological outcomes (0–10)	Clinical outcomes (0–5)	Assessment of clinical outcomes (0–9)	Patient selection process (0–11)	Total score (0–100)
Cobianchi Bellisari et al. (2021)	4	0	10	0	5	2	10	0	5	2	6	44
Van Genechten et al. (2021)	7	2	4	10	5	0	0	0	5	5	8	46
Ostojic et al. (2024)	4	0	4	10	3	5	0	0	5	5	8	44
Pintat et al. (2017)	0	2	4	10	5	0	10	0	5	5	6	47
Guimarães et al. (2018)	0	2	4	10	5	0	10	0	5	5	8	49
Vasso et al. (2022)	4	2	4	0	5	5	0	0	5	5	6	36
Silvestre et al. (2023)	10	2	10	0	5	2	10	0	5	5	6	55

MRI: magnetic resonance imaging.

### 3.2 Patients’ demographics

The four studies assessing PRP-based therapies (Örsçelik et al., 2020; Cobianchi et al., 2021; Van et al., 2021; Ostojic et al., 2024) included a total of 142 patients, with sample sizes ranging from 26 to 50 subjects. The mean age of participants ranged from 34.6 to 50.4 years old. Sex distribution varied across the included articles, although Van et al. (2021) exclusively enrolled female patients. Body mass index (BMI) was inconsistently reported and, when available, generally indicated overweight populations. Follow-up duration ranged from 6 to 17 months, and most patients presented with moderate-to-severe PFC or PFOA (according to the McCauley classification, Outerbridge classification, and Patello-Femoral Cartilage Wear).

On the other hand, the remaining five studies evaluating MSC-based products (Pintat et al., 2017; Guimarães et al., 2018; Vasso et al., 2022; Silvestre et al., 2023; Zhang et al., 2024) involved a total of 181 patients, with sample sizes ranging from 8 to 96 subjects. The mean age of participants ranged from 43.1 to 58 years old. Overall, both sexes were well represented across studies, although with variable distribution, and BMI was inconsistently reported. The included populations mainly consisted of subjects affected by moderate-to-severe PFOA (according to the International Cartilage Repair Society-like classification, Outerbridge classification, Iwano classification, and Kellgren-Lawrence classification), with follow-up periods ranging from 12 to 24 months.

### 3.3 PRP-based products

Four studies (Örsçelik et al., 2020; Cobianchi et al., 2021; Van et al., 2021; Ostojic et al., 2024) evaluated the therapeutic efficacy of PRP-based products administered by intra-articular injection in the treatment of PFC or PFOA. More in detail, three studies investigated PRP (used in combination with a standard 12-week exercise program by Örsçelik et al. (2020), HA by Ostojic et al. (2024), and alone by Cobianchi et al. (2021)), whereas the fourth and last study analyzed the application of APS (used alone by Van et al. (2021)). Örsçelik et al. (2020) and Cobianchi et al. (2021) employed ultrasound guidance to verify the effectiveness of the intra-articular injection. Cobianchi et al. (2021) and Van et al. (2021) also assessed radiological outcomes. Study designs were as follows: one RCT (Örsçelik et al., 2020), one retrospective study (Cobianchi et al., 2021), and two prospective studies (Van et al., 2021; Ostojic et al., 2024). Clinical and, where reported, radiological findings are summarized in Table 2 for each study, whereas the type of product employed, the preparation method/kit, the injected volume, the number of platelets, and the injection protocol are outlined in Table 3.

**TABLE 2:** Studies evaluating the use of platelet-rich plasma-based products in the treatment of patello-femoral chondropathy or osteoarthritis.

Publication	Study design	Level of evidence	Patients’ characteristics	PFC/PFOA grade	Therapeutic protocol	Outcome measures	Follow-up	Clinical findings	Radiological findings
Örsçelik et al. (2020)	RCT	I	N° of cases: 38 Sex: 16 F, 22 M Age: 44.5 ± 10.3 years BMI: N/A	15 grade II, 16 grade III, 7 grade IV (McCauley classification)	Three, US-guided IA injections of PRP vs. PrT at 3-week intervals + standard 12-week exercise program	VAS, TLKSS	17.1 ± 5.4 months	Clinical superiority of PRP compared to PrT in terms of VAS, TLKSS, pain during exercise, ROM, crepitus, and total number of medications; 1 drop-out and 5 lost to follow-up in the PrT group	–
Cobianchi Bellisari et al. (2021)	Retrospective case-control study	III	N° of cases: 34 Sex: 12 F, 22 M Age: 41.8 ± 8.9 years BMI: <30 kg/m^2^	4 grade II, 18 grade III, 4 grade IV (Outerbridge classification)	Three, US-guided IA injections of PRP at 3-week intervals vs. conservative treatment	VAS, WOMAC, MRI	6.4 ± 1.9 (range 4–12) months	Clinical superiority of PRP compared to conservative treatment in terms of VAS and WOMAC	Statistically significant improvement of T^2^ relaxation times compared to baseline in the PRP group
Van Genechten et al. (2021)	Prospective case series	IV	N° of cases: 50 Sex: 50 F Age: 50.4 ± 6.5 years BMI: 26.9 ± 4.3 kg/m^2^	14% mild, 24% moderate, 62% severe (PFCW)	Single or two (in 28/50 patients) IA injections of APS at 3-month intervals	KOOS, NRS, Kujala, UCLA, EQ-5D, MRI	12 months	Response rate 53.7% at 12 months; statistically significant improvement of KOOS pain, NRS, and Kujala compared to baseline; second injection after 3 months showed little efficacy in initially poor responding patients; only female patients; 9 drop-out	Patients with major synovitis on MRI improved more
Ostojic et al. (2024)	Prospective cohort study	III	N° of cases: 28 (2 patients with both knees) Sex: 20 F, 10 M Age: 34.6 ± 9.9 years BMI: N/A	N/A	Three IA injections of PRP at 7–10-day intervals + single IA injection of HA vs. physiotherapy alone	Kujala, VAS	6 months	Clinical superiority of PRP + HA compared to physiotherapy alone in terms of Kujala and VAS; younger patients seemed to benefit more in the injection group	–

PFC: patello-femoral chondropathy; PFOA: patello-femoral osteoarthritis; RCT: randomized controlled trial; F: females; M: males; BMI: body mass index; N/A: not applicable; US: ultrasound; IA: intra-articular; PRP: platelet-rich plasma; PrT: prolotherapy; VAS: visual analogue scale; TLKSS: Tegner-Lysholm Knee Scoring Scale; ROM: range of motion; WOMAC: Western Ontario McMaster Universities Osteoarthritis Index; MRI: magnetic resonance imaging; PFCW: Patello-Femoral Cartilage Wear; APS: autologous protein solution; KOOS: knee injury and osteoarthritis outcome score; NRS: numerical rating scale; EQ-5D: European Quality of Life 5 Dimension; HA: hyaluronic acid.

**TABLE 3:** Summary of the type of product employed, the preparation method/kit, the injected volume, the number of platelets, and the injection protocol of the studies evaluating the use of platelet-rich plasma-based products in the treatment of patello-femoral chondropathy or osteoarthritis.

Publication	Product	Preparation method/Kit	Injected volume	Number of platelets	Injection protocol
Örsçelik et al. (2020)	PRP	72 mL of venous blood +8 mL of citrate centrifuged at 3200 rpm for 15 min *GPS III Platelet Concentration System (Biomet Biologics)*	∼7 mL (+1 mL used to calculate platelet concentration)	–	Three, US-guided IA injections of PRP at 3-week intervals + standard 12-week exercise program
Cobianchi Bellisari et al. (2021)	PRP	Activated with CaCl_2_ (1:20) and buffered with NaCl (1:10)	∼8–10 mL	–	Three, US-guided IA injections of PRP at 3-week intervals
Van Genechten et al. (2021)	APS	Cell-separating component (that splits the cellular components of plasma from RBCs) and an APS concentrating component (that filters plasma by polyacrylamide beads) *nSTRIDE® APS Kit (Zimmer Biomet)*	∼2.5 mL	–	Single or two IA injections of APS at 3-month intervals
Ostojic et al. (2024)	PRP	15 mL of venous blood *Arthrex ACP System (Arthrex)*	∼4–6 mL	–	Three IA injections of PRP at 7–10-day intervals + single IA injection of HA

PRP: platelet-rich plasma; rpm: revolutions per minute; US: ultrasound; IA: intra-articular; APS: autologous protein solution; RBCs: red blood cells; HA: hyaluronic acid.

In 2020, a RCT conducted by Örsçelik et al. (2020) examined the therapeutic efficacy of three, ultrasound-guided intra-articular injections of PRP versus prolotherapy administered at 3-week intervals combined with a standard 12-week exercise program in the treatment of 75 patients (38 cases and 37 controls) with persistent symptomatic chondromalacia patellae after failure of at least 3 months of conservative management. Relative to the control group, they noticed a clinical superiority of PRP at all time-points in terms of Visual Analogue Scale (VAS) scale, Tegner-Lysholm Knee Scoring Scale (TLKSS) score, pain during exercise, range of motion, crepitus, and total number of medications (p = 0.001, p = 0.026, p = 0.004, p = 0.038, p < 0.001, and p = 0.003, respectively). Of note, the statistical power of the above-mentioned findings was limited by the withdrawal of one patient from the control group after the first injection as well as by the loss to follow-up of five additional participants from the same group.

In 2021, Cobianchi et al. (2021) published a retrospective case-control study on the use of three, ultrasound-guided intra-articular injections of PRP administered at 3-week intervals versus conservative management in the treatment of 68 patients (34 cases and 34 controls) affected by PFC. Relative to the control group, they recorded a statistically significant improvement of the VAS scale and Western Ontario McMaster Universities Osteoarthritis Index (WOMAC) score from baseline to the latest follow-up (from 18.3 ± 4.5 to 7.3 ± 3.2 and from 7 to 2, respectively; p < 0.001), confirming the clinical superiority of PRP compared to conservative treatment. As it pertains T^2^ relaxation times, in the PRP group, they reported a statistically significant amelioration from baseline to the latest follow-up considering all four compartments – that is, medial and lateral femoral condyle, medial and lateral patella –, the index compartment – that is, the compartment with the highest modified Whole-Organ Magnetic Resonance Score (WORMS) values –, and focal articular cartilage lesions (from 44.2 ± 2.5 ms to 41.5 ± 2.5 ms, from 47.8 ± 3.6 ms to 43.5 ± 3.8 ms, and from 70.1 ± 13.0 ms to 59.9 ± 4.6 ms, respectively; p < 0.001).

In 2021, Van et al. (2021) performed a prospective case series analyzing the application of a single or two (in 28/50 participants) intra-articular injections of APS administered at 3-month intervals in the treatment of 50 female patients with mainly moderate-to-severe (86%) patello-femoral cartilage wear. They found that the response rate remained stable throughout the study period, reaching 53.7% at the 12-month follow-up. Moreover, they described a statistically significant improvement of the Knee Injury and Osteoarthritis Outcome Score (KOOS) pain score, Numerical Rating Scale (NRS) scale, and Kujala score at 12 months compared to baseline (from 40.3 ± 18.7 to 57.3 ± 24.8, from 6.7 ± 2.2 to 4.5 ± 2.9, and from 48.4 ± 13.0 to 56.3 ± 18.1, respectively; p < 0.05). On the contrary, the UCLA score and European Quality of Life 5 Dimension (EQ-5D) score demonstrated no statistically significant differences at any post-injection follow-up time-point. Interestingly, subjects with evidence of major synovitis on magnetic resonance imaging (MRI) appeared to improve more on the KOOS pain score, KOOS symptoms score, and KOOS activities of daily living score than those with no-minor synovitis. For the 28 participants who requested it, a second intra-articular injection of APS after 3 months revealed a stabilization of the response rate, with limited effectiveness in initially poor responding patients. Over the course of the study period, nine subjects (18%) were excluded from subsequent analysis after undergoing alternative injection therapies or surgery at the index knee.

Finally, in 2024, Ostojic et al. (2024) conducted a prospective cohort study on the use of three intra-articular injections of PRP administered at 7–10-day intervals combined with a single intra-articular injection of HA (Synolis 2%) versus physiotherapy alone in the treatment of 43 patients (28 cases and 15 controls) affected by chondromalacia patellae. Relative to the control group, they described a clinical superiority of PRP plus HA at all time-points in terms of Kujala score and VAS scale (p < 0.001), however, no statistically significant differences were reported between the 3- and 6-month follow-up. Notably, in the injection group, younger subjects seemed to derive greater benefit from treatment than elderly patients, as evidenced by the more pronounced improvement in the Kujala score and VAS scale associated with decreasing age.

### 3.4 MSC-based products

Five studies (Pintat et al., 2017; Guimarães et al., 2018; Vasso et al., 2022; Silvestre et al., 2023; Zhang et al., 2024) assessed the therapeutic efficacy of MSC-based products administered by intra-articular injection in the treatment of PFOA. More in detail, two studies investigated SVF (used in combination with PRP by Pintat et al. (2017) and alone by Zhang et al. (2024)), whereas the remaining three studies examined the application of BMMCs (used in combination with arthroscopy by Guimarães et al. (2018)), BMC (used alone by Silvestre et al. (2023)), and MFAT (used in combination with arthroscopy by Vasso et al. (2022)). Pintat et al. (2017) employed fluoroscopy guidance to verify the effectiveness of the intra-articular injection, whereas Silvestre et al. (2023) used ultrasound guidance for the same purpose. Pintat et al. (2017), Guimarães et al. (2018), Silvestre et al. (2023), and Zhang et al. (2024) also considered radiological outcomes. Study designs were as follows: two prospective studies (Pintat et al., 2017; Guimarães et al., 2018), two retrospective studies (Vasso et al., 2022; Silvestre et al., 2023), and one RCT (Zhang et al., 2024). Clinical and, where reported, radiological findings are summarized in Table 4 for each study, whereas the type of product employed, the preparation method/kit, the injected volume, the number of cells, and the injection protocol are outlined in Table 5.

**TABLE 4:** Studies evaluating the use of mesenchymal stromal cell-based products in the treatment of patello-femoral osteoarthritis.

Publication	Study design	Level of evidence	Patients’ characteristics	PFOA grade	Therapeutic protocol	Outcome measures	Follow-up	Clinical findings	Radiological findings
Pintat et al., 2017 (43)	Prospective cohort study	IV	N° of cases: 19 Sex: 9 F, 10 M Age: 43.1 y BMI: N/A	Global grade 2.7 ± 1 (ICRS-like classification)	Single, fluoroscopy-guided IA injection of SVF + PRP	WOMAC, MRI	12 months	Statistically significant improvement of WOMAC compared to baseline; 4 lost to follow-up	No relevant changes on MRI
Guimarães et al., 2018 (44)	Prospective pilot study	IV	N of cases: 8 Sex: 4 F, 4 M Age: 52.5 years BMI: N/A	8 grade IV (Outerbridge classification)	Single IA injection of BMMCs + arthroscopy	TLKSS, SF-36, MRI	24 months	Statistically significant improvement of TLKSS and SF-36 compared to baseline; no statistically significant differences between the 12- and 24-month follow-up	Notable improvement of the patello-femoral chondral cap on MRI in 6/8 patients
Vasso et al., 2022 (45)	Retrospective case series	IV	N of cases: 23 Sex: 15 F, 8 M Age: 58 ± 8 years BMI: 28.0 ± 4.8 kg/m^2^	5 grade I, 11 grade II, 7 grade III (Iwano classification)	Single IA injection of MFAT + arthroscopy	IKS, VAS	22.1 ± 4.2 (range, 15–30) months	Statistically significant improvement of IKS and VAS compared to baseline; improvements were not affected by age, BMI, or PFOA grade; satisfactory restoration of stair-climbing ability in 21/23 patients	–
Silvestre et al., 2023 (46)	Retrospective case-control study	IV	N of cases: 96 Sex: 33 F, 66 M Age: 53 ± 13 years BMI: 25.5 ± 3.3 kg/m^2^	N/A	Single, US-guided IA injection of BMC vs. conservative treatment	IKDC, WOMAC, VAS, MRI	12 months	Clinical superiority of BMC compared to conservative treatment in terms of IKDC, WOMAC, and VAS	Stabilization of articular cartilage volume on MRI in the SVF group
Zhang et al., 2024 (47)	RCT	I	N of cases: 35 Sex: 27 F, 8 M Age: 57.6 ± 12.3 years BMI: 24.1 ± 2.8 kg/m^2^	6 grade I, 15 grade II, 14 grade III (K-L classification); 21 grade I, 9 grade II, 5 grade III (Iwano classification)	Three IA injections of SVF vs. HA at 1-month intervals	VAS, WOMAC, SMCPS, MRI	12 months	Clinical superiority of SVF compared to HA in terms of VAS, WOMAC, and SMCPS	Improvement of both the articular cartilage and bone marrow score of the PF joint and only of the bone marrow score of the TF joint on MRI in the SVF group

PFOA: patello-femoral osteoarthritis; F: females; M: males; BMI: body mass index; ICRS: international cartilage repair society; IA: intra-articular; SVF: stromal vascular fraction; PRP: platelet-rich plasma; WOMAC: Western Ontario McMaster Universities Osteoarthritis Index; MRI: magnetic resonance imaging; BMMCs: bone marrow-derived mononuclear cells; TLKSS: Tegner-Lysholm Knee Scoring Scale; SF-36: Short Form 36; MFAT: micro-fragmented adipose tissue; IKS: international knee society; VAS: visual analogue scale; N/A: not applicable; US: ultrasound; BMC: bone marrow concentrate; IKDC: international knee documentation committee; RCT: randomized controlled trial; K-L: Kellgren-Lawrence; HA: hyaluronic acid; SMCPS: Samsung Medical Center Patello-femoral Score; PF: patello-femoral; TF: tibio-femoral.

**TABLE 5:** Summary of the type of product employed, the preparation method/kit, the injected volume, the number of cells, and the injection protocol of the studies evaluating the use of mesenchymal stromal cell-based products in the treatment of patello-femoral osteoarthritis.

Publication	Product	Preparation method/kit	Injected volume	Number of cells	Injection protocol
Pintat et al. (2017)	SVF + PRP	Autologous adipose tissue processed via enzymatic digestion (collagenase); PRP prepared using the *Arthrex ACP System*	∼5 mL (total)	3.9 × 10^6^/mL of adipose tissue-derived MSCs suspended in autologous PRP	Single, fluoroscopy-guided IA injection of SVF + PRP
Guimarães et al. (2018)	BMMCs	Centrifugation using a specific density gradient (Ficoll-Paque)	∼5 mL	5.6 × 10^7^/mL (viability >95%)	Single IA injection of BMMCs + arthroscopy
Vasso et al. (2022)	MFAT	Mechanical manipulation using the *Lipogems System*	∼6–9 mL	–	Single IA injection of MFAT + arthroscopy
Silvestre et al. (2023)	BMC	Rapid centrifugation using the *MarrowSonic device*	∼6 mL	–	Single, US-guided IA injection of BMC
Zhang et al. (2024)	SVF	Autologous adipose tissue processed via enzymatic digestion (collagenase NB4)	∼4 mL	3.78 × 10^7^/injection	Three IA injections of SVF vs. HA at 1-month intervals

SVF: stromal vascular fraction; PRP: platelet-rich plasma; MSCs: mesenchymal stromal cells; IA: intra-articular; BMMCs: bone marrow-derived mononuclear cells; MFAT: micro-fragmented adipose tissue; BMC: bone marrow concentrate; US: ultrasound; HA: hyaluronic acid.

In 2017, Pintat et al. (2017) published a prospective cohort study on the use of a single, fluoroscopy-guided intra-articular injection of autologous SVF and PRP in the treatment of 19 patients with persistent symptomatic PFOA after failure of initial conservative management. They documented a statistically significant amelioration of the WOMAC score at 6 and 12 months compared to baseline (from 34.3 ± 24 to 14.1 ± 14.2; p < 0.0018). As similarly highlighted by Örsçelik et al. (2020), the statistical power of the above-mentioned findings was limited by the small sample size as well as by the loss to follow-up of four participants by the end of the 12-month follow-up. Concerning the 6-month MRI follow-up, no relevant changes were observed compared to baseline in terms of lesion grade, size, or T2 value (p > 0.375).

In 2018, a prospective pilot study conducted by Guimarães et al. (2018) assessed the therapeutic efficacy of a single intra-articular injection of autologous BMMCs combined with arthroscopic debridement and lavage in the treatment of eight patients with persistent symptomatic PFOA after failure of at least 1 year of conservative management with oral NSAIDs and physiotherapy. They recorded a statistically significant improvement of the TLKSS score at 12 months compared to baseline (from 48.3 to 97.3; p < 0.001), however, no statistically significant differences were noted between the 12- and 24-month follow-up (p > 0.10). Additionally, they observed a statistically significant amelioration of the Short Form 36 (SF-36) scale at 12 months compared to baseline for all domains (p < 0.001), again, with no statistically significant differences between the 12- and 24-month follow-up in all domains but general state of health and mental health (p < 0.01). Interestingly, six out of eight patients displayed a notable improvement of the patello-femoral chondral cap on MRI.

In 2022, Vasso et al. (2022) performed a retrospective case series on 23 patients affected by early-to-moderate PFOA who were treated with a single intra-articular injection of autologous MFAT combined with arthroscopic debridement (i.e., chondral shaving or abrasion and/or meniscal regularization depending on the type of lesion identified). They described a statistically significant improvement of the International Knee Society (IKS) knee score, IKS function score, and VAS scale from baseline to the latest follow-up (from 35.6 ± 14.9 to 61.9 ± 17.8, from 52.0 ± 14.7 to 82.3 ± 19.1, and from 8.7 ± 2.2 to 5.i2 ± 2.5, respectively; p < 0.001), regardless of patients’ age, BMI, or PFOA grade. Furthermore, a satisfactory restoration of stair-climbing ability was found in 21 out of 23 patients at the latest follow-up. Nonetheless, the authors did not specify whether outcomes differed according to the type of arthroscopic intervention performed, which, unfortunately, limits the interpretation of the potential influence of concomitant surgical procedures.

In 2023, Silvestre et al. (2023) published a retrospective case-control study analyzing the use of a single, ultrasound-guided intra-articular injection of autologous BMC versus conservative management with oral NSAIDs and physiotherapy in the treatment of 117 patients (96 cases and 21 controls) with persistent symptomatic PFOA after failure of initial conservative management. Relative to the control group, they found a statistically significant amelioration of the International Knee Documentation Committee (IKDC) score, WOMAC score, and VAS scale at 12 months compared to baseline (from 42.0 ± 13.7 to 58.6 ± 16.7, from 37.2 ± 18.5 to 22.8 ± 18.5, and from 5.4 ± 1.8 to 3.6 ± 2.3, respectively; p < 0.0001), confirming the clinical superiority of BMC compared to conservative treatment. Of note, in the injection group, they identified a trend towards a stabilization of the articular cartilage volume, with a mean loss of about 0.7% at 12 months compared to a mean loss of about 6.9% in the control group.

Lastly, in 2024, Zhang et al. (2024) conducted a RCT comparing the application of three intra-articular injections of autologous SVF versus HA administered at 1-month intervals in the treatment of 70 patients (35 cases and 35 controls) affected by multi-compartmental knee OA. In the SVF group, the VAS scale, WOMAC score, and Samsung Medical Center Patello-femoral Score (SMCPS) scores showed a statistically significant amelioration at 3, 6, and 12 months compared to baseline. In the HA group, the VAS scale and WOMAC score improved at 3 and 6 months but returned to the pre-injection levels by 12 months with no statistically significant difference (p > 0.05), whereas the SMCPS scores remained stable at 3 months and increased only at 6 and 12 months compared to baseline. Between-group comparisons revealed that the SVF group had a significantly better VAS scale than the HA group at 6 and 12 months, a better WOMAC score only at 6 months, and better SMCPS scores at all time-points, confirming the clinical superiority of SVF compared to HA. Notably, since the WOMAC score reflects the status of the entire knee – including both the tibio-femoral and patello-femoral joint – and the SMCPS scores represent the function only of the patello-femoral compartment, these findings indicate that the tibio-femoral and patello-femoral joint do not respond to SVF in the exact same way. In accordance with the above-mentioned clinical results, in the SVF group, the patello-femoral joint exhibited an improvement of both the articular cartilage and bone marrow score on MRI at 12 months compared to baseline, whereas the tibio-femoral joint showed an amelioration only of the bone marrow score. In contrast, in the control group, none of the afore-mentioned scores improved from baseline.

## 4 Discussion

The present systematic review aimed to critically appraise the available clinical and, where reported, radiological evidence on the use of PRP- and MSC-based products in the treatment of PFC and PFOA. Overall, our findings highlighted a growing interest in orthobiologic approaches for the management of patello-femoral pathology; however, they also revealed the presence of major methodological and evidentiary limitations in the existing literature that currently preclude the formulation of strong, evidence-based recommendations guiding their application in the routine clinical practice.

Our principal finding is the generally poor methodological quality of the existing body of evidence. Most of the included articles were retrospective or uncontrolled investigations with small sample sizes (and, indeed, the largest population was 96 participants in the study by Silvestre et al. (2023)), short- or medium-term follow-up times (and, indeed, the longest follow-up period was 22.1 ± 4.2 months in the study by Vasso et al. (2022)), and an overall high risk of bias, as reflected by the presence of some concerns overall in the two RCTs (Örsçelik et al., 2020; Zhang et al., 2024) and the low modified CMS of the seven non-RCTs (Cobianchi et al., 2021; Van et al., 2021; Ostojic et al., 2024; Pintat et al., 2017; Guimarães et al., 2018; Vasso et al., 2022; Silvestre et al., 2023) included in the present analysis. Another critical issue emerging from this systematic review is the pronounced heterogeneity across the included studies. Significant variability was reported in terms of patient selection (with patello-femoral pathology ranging from isolated chondromalacia patellae to advanced PFOA), grading systems, and patients’ characteristics. Similarly, also the types of products employed differed widely in terms of biological source, processing techniques, cellular composition, and final formulation, particularly among MSC-based therapies. Injection protocols exhibited considerable differences in terms of number of injections, dosing intervals, image guidance modalities, and combination with adjunctive treatments like arthroscopy, standardized exercise programs, or HA. Lastly, in many studies, the statistical power of the results was further undermined by losses to follow-up, incomplete reporting of patients’ characteristics, or concomitant surgical procedures, which are all important confounding factors hindering the comparability of outcomes and, consequently, preventing meaningful quantitative synthesis. Taken together, all these methodological caveats contribute to limit the strength of the available clinical evidence, hampering the ability to draw definitive conclusions on the actual therapeutic efficacy of PRP- and MSC-based approaches for patello-femoral pathology.

Despite the afore-mentioned limitations, a consistent trend towards a clinical improvement was observed across the included studies. Both PRP- and MSC-based interventions were in most cases associated with a marked reduction in pain scores and considerable improvements in patient-reported outcome measures at both short- and medium-term follow-up times, while demonstrating a favourable safety profile. Although between-group comparisons confirmed the clinical superiority of both PRP- and MSC-based approaches compared to the controls (PRP versus prolotherapy (Örsçelik et al., 2020), PRP versus conservative treatment (Cobianchi et al., 2021), PRP plus HA versus physiotherapy alone (Ostojic et al., 2024), BMC versus conservative treatment (Silvestre et al., 2023), and SVF versus HA (Zhang et al., 2024)), none of the included articles directly compared the performance of these two orthobiologic strategies, underscoring the need for well-designed comparative studies to clarify their respective roles in the management of patello-femoral joint disorders. Interestingly, some authors also suggested a potential biological effect of orthobiologics on the articular cartilage tissue itself of the patello-femoral compartment, as supported by MRI findings such as stabilization of the articular cartilage volume, improvements in T2 relaxation times, and amelioration of both the articular cartilage and bone marrow score (Cobianchi et al., 2021; Van et al., 2021; Guimarães et al., 2018; Silvestre et al., 2023; Zhang et al., 2024). While these imaging results should be interpreted with caution, they support the hypothesis that PRP- and MSC-based products may exert some disease-modifying effects in addition to a symptomatic relief also in the treatment of patello-femoral pathology (Perucca et al., 2023; Boffa et al., 2023; Boffa et al., 2021).

The encouraging results reported in the present systematic review are consistent with findings from the broader body of literature on the use of orthobiologics in the treatment of tricompartmental knee OA. To this regard, in the past 2 years, the ESSKA Orthobiologic Initiative has published a series of two consensuses on the use of blood- (Laver et al., 2024) and cell-based products (De et al., 2025) for the intra-articular injective treatment of knee OA. More in detail, the former concluded that there are enough pre-clinical and clinical data to support the use of PRP-based interventions as a first-line injectable for mild-to-moderate knee OA (that is, Kellgren-Lawrence grade I-III), considering its more durable effects and better safety profile compared to corticosteroids, whereas the latter recommended the selection of cell-based therapies as a second-line injectable for mild-to-moderate and, possibly, severe knee OA (that is, Kellgren-Lawrence grade IV), especially after failure of conventional conservative treatments. Importantly, the consensus group confirmed that both blood- and cell-based products are a clinically better injectable option than HA; additionally, it concluded that, based on the existing body of evidence, only PRP- but not MSC-based interventions have demonstrated clinical superiority compared to corticosteroids; and, lastly, it did not acknowledge any clear advantage of cell-based approaches over blood-based products nor from their combined use. That being said, clinical evidence derived from tricompartmental knee OA is not directly generalizable to patello-femoral pathology, since, as previously discussed, the patello-femoral joint constitutes a unique biomechanical and biological environment characterized by a complex, multi-planar kinematics, high contact loads, and considerable shear stresses that may negatively impact the therapeutic response to PRP- and MSC-based therapies. In fact, clinical data supporting the use of orthobiologics in the initial management of patello-femoral pathology remains inadequate, as all the analyses published so far evaluate the knee as a whole or choose to focus on a single compartment (only tibio-femoral or patello-femoral) without directly comparing the two. To date, the only high-quality clinical evidence on the topic is limited to a single systematic review published by Chalidis et al. (2025) in 2025 on the use of PRP-based approaches in the treatment of chondromalacia patellae, PFOA, or anterior knee pain. More precisely, they considered five studies (Örsçelik et al., 2020; Cobianchi et al., 2021; Ostojic et al., 2024; Pintat et al., 2017; Gürsoy et al., 2025) and concluded that, despite the lack of specific recommendations guiding their use in the routine clinical practice, PRP-based products appear to be a safe alternative with promising therapeutic benefits also in the treatment of patello-femoral pathology. Regarding MSC-based interventions, we found no systematic reviews nor metanalyses in the literature investigating their application for patello-femoral joint disorders, confirming the above-mentioned unmet need for compartment-specific investigations to elaborate tailored treatment strategies.

Taken together, the existing research suggests that PRP- and MSC-based therapies hold promise for the management of PFC and PFOA, particularly in patients who have failed initial conservative management. Nevertheless, the paucity of high-quality RCTs, the lack of standardized treatment protocols, and the scarcity of long-term follow-up data remain a critical gap in the current literature. Thus, future research should prioritize well-designed RCTs with adequate sample sizes, clear inclusion criteria focused on patello-femoral pathology, standardized orthobiologic formulations, and uniform outcome measures to establish clear indications, define treatment protocols, and, ultimately, investigate the durability of the therapeutic effects of PRP- and MSC-based products in the treatment of PFC and PFOA.

## 5 Conclusion

The present systematic review demonstrated that the available clinical and, where reported, radiological evidence on PRP- and MSC-based products for patello-femoral joint disorders remains limited and methodologically heterogeneous, preventing the elaboration of strong, evidence-based recommendations for their application in the routine clinical practice. Nonetheless, the majority of studies published in the existing literature report encouraging results in terms of pain relief, functional improvements, and volumetric MRI findings as well as an acceptable safety profile, proving that, with careful patient selection and appropriate counselling, PRP- and MSC-based products may hold a therapeutic potential for PFC and PFOA, extending beyond their established application for tricompartmental knee OA.

## Data Availability

The original contributions presented in the study are included in the article/supplementary material, further inquiries can be directed to the corresponding author.
